# Study of Antibiotic Susceptibility among Bacterial Isolates in Neonatal Intensive Care Unit of a Tertiary Care Hospital: A Descriptive Cross-sectional Study

**DOI:** 10.31729/jnma.5216

**Published:** 2020-11-30

**Authors:** Brajesh Raj Chaudhary, Kalpana Karmacharya Malla, Sajan Poudel, Brajesh Kumar Jha

**Affiliations:** 1 Department of Pediatrics, College of Medical Sciences and Teaching Hospital, Bharatpur, Nepal; 2 Department of Microbiology, College of Medical Sciences and Teaching Hospital, Bharatpur, Nepal

**Keywords:** *antibiotic resistance*, *clinico-bacteriological profile*, *neonatal sepsis*

## Abstract

**Introduction:**

Neonatal sepsis is a major cause of neonatal morbidity and mortality worldwide, especially in developing countries like Nepal. Antibiotic resistance among microorganisms poses new challenges in the treatment of neonatal sepsis. The present study is conducted with the objectives of determining clinico-bacteriological profile and antibiotic susceptibility among isolated bacteria in a neonatal intensive care unit.

**Methods:**

A descriptive cross-sectional study was conducted from January 1, 2017, to December 31, 2019, in the neonatal intensive care unit of a tertiary care hospital after obtaining ethical clearance from Institutional Review Committee (Ref: 2020-064). The sample size was calculated and 77 neonates with culture-proven sepsis were included in the study. The antibiotic susceptibility tests of the isolates were done by Kirby-Bauer disc diffusion method. Data entry was done in Statistical Packages for the Social Sciences version 20.

**Results:**

Of the 841 specimens (blood, cerebrospinal fluid, urine, tracheal aspirate and pus) processed for culture, bacteria were isolated in 84 (10.0%) specimens. Among the 84, gram-negative bacilli were the predominant isolates 76 (90.5%); of which *Acinetobacter baumannii* was the most common 27 (32.1%). Both the Gram-negative and the Gram-positive bacteria showed high resistance to Penicillin and Cephalosporins. Gram-negative bacteria showed maximum sensitivity to Colistin, Carbapenems, Tigecycline and Fluoroquinolones. Gram-positive bacteria showed maximum susceptibility to Amikacin, Vancomycin and Carbapenems.

**Conclusions:**

Judicious use of antibiotics based on the updated knowledge of prevalent organisms in the local hospital setting and their antibiotic sensitivity pattern is of utmost importance for the effective treatment of neonatal sepsis.

## INTRODUCTION

Neonatal sepsis remains one of the leading causes of morbidity and mortality worldwide, especially in developing countries like Nepal.^1–3^ According to Nepal Demographic and Health Survey (2016), the neonatal mortality rate in Nepal is 21/1000 live births, the major portion of which is constituted by neonatal sepsis (16.0%).^3^

Antibiotic resistance has become a global problem. The increasing trend of multidrug-resistant bacteria causing neonatal sepsis in developing countries, particularly in intensive care, poses new challenges in their treatment. Premature babies, those receiving mechanical ventilation, intravenous fluids, central lines, and prolonged hospital stay, are at major risk.^4–6^

The updated knowledge about antibiotics susceptibility pattern among the microorganisms in hospitals is important for the effective treatment of neonatal sepsis. Hence, the present study is conducted with the aim of determining clinico-bacteriological profile and antibiotic susceptibility pattern among the isolated bacteria in the neonatal intensive care unit of a tertiary care hospital in Nepal.

## METHODS

This descriptive cross-sectional study was conducted in the neonatal intensive care unit (NICU) of College of Medical Sciences and Teaching Hospital, Bharatpur, Nepal over a period of three years from January 1, 2017, to December 31, 2019, after obtaining ethical approval from Institutional Review Committee (Reference Number: 2020-064). Of all the cases of clinically suspected neonatal sepsis admitted in the NICU, only neonates with culture-proven sepsis were included in the study. Convenient sampling was done. The sample size was calculated using the formula,

$$\begin{array}{ll} \text{n} & \text{= }{\text{Z}}^{\text{2}}\times \text{p}\times \text{(1}-\text{p)}/{\text{e}}^{\text{2}}\\ & \text{= }{\left(1.96\right)}^{\text{2}}\times 0.04\times 0.96/{(\text{0.05)}}^{\text{2}}\\ & \text{= }59 \end{array}$$

Where,

n = sample size
Z = 1.96 at 95% Confidence Interval (CI)
p = past prevalence, 4%^7^
e = margin of error, 5%

During the study period, 77 neonates were enrolled which was adequate for the study. Clinical suspicion of neonatal sepsis was based on the manifestations such as respiratory distress, temperature instability, poor feeding, poor cry, lethargy, cyanosis, bleeding, hypoglycemia, apnea, and seizure either at the time of admission or during the course of stay in NICU. The neonates having symptoms within 72 hours of life were defined as early-onset neonatal sepsis (EONS) and those having symptoms after 72 hours of life were defined as late-onset neonatal sepsis (LONS).^8^

Blood culture was performed in all the clinically suspected cases of sepsis, while cultures of cerebrospinal fluid (CSF), tracheal aspirate, urine and pus were done when required. All specimens were collected undertaking standard aseptic precaution. For blood culture, 1-2 ml of the blood sample was obtained and inoculated immediately in Brain Heart Infusion broth (HiMedia, M20) in a ratio of 1:5. The culture bottle was incubated at 37°C and all the specimens were blindly subcultured after 18 hrs and 48 hrs on MacConkey agar, blood agar, and chocolate agar. The culture specimen which did not show any growth was reincubated till 7th day. Other specimen were also cultured and Identified as per standard microbiological techniques, which involved morphological appearance of the colonies, Gram's staining reaction, and various biochemical properties (Catalase test, Coagulase test, Oxidase test, Triple Sugar Iron agar (TSI) media, Sulphide Indole Motility (SIM) media, Simmon's Citrate media, Chirstensen's Urea media, Methyl Red/Voges Proskauer (MR/VP) media.

The antibiotic sensitivity tests of the isolates were done using Muller Hinton Agar (MHA) (HiMedia, India) by Kirby- Bauer disc diffusion method as per the recommendation of Clinical and Laboratory Standards Institute (CLSI).^9^ In our study, Amikacin (10 mcg), Ampicillin (25 mcg), Azithromycin (30 mcg), Cefepime (50 mcg), Cefoperazone/Sulbactam (75/30), Ceftriaxone (10 mcg), Cefotaxime (10 mcg), Cotrimoxazole (25 mcg), Chloramphenicol (25 mcg), Colistin (25 mcg), Ofloxacin (5 mcg), Meropenem (10 mcg), Gentamicin (50 mcg), Norfloxacin (5 mcg), Piperacillin/Tazobactam (100/10 mcg), Ampicillin/Sulbactam (10/10 mcg), Penicillin (10 units), Polymixin B (50 units), Cloxacillin (1 mcg), Imipenem (10 mcg) and Vancomycin (30 mcg) discs (HiMedia) were used. Control strains of E. coli ATCC 25922, S. aureus ATCC 25923, P. aeruginosa ATCC 27853, and K. pneumoniae ATCC 700603 were used for standardization and correct interpretation of zone of diameter.

The data was entered and analyzed using Statistical Package for the Social Sciences (SPSS) version 20.

## RESULTS

Out of 687 neonates with clinical suspicion of sepsis, 77 (11.2%) showed culture positivity; 70 (10.2%) had mono-microbial and 7 (1.0%) had polymicrobial sepsis. Among culture-positive neonates, males were more common than the female with a ratio of 2.2:1. Neonates delivered by caesarean section were 33 (42.9%), while preterm babies constituted 19 (24.7%). Early-onset neonatal sepsis (EONS) was observed in 66.2% of cases. Very low birth weight (< 1500gm) and low birth weight(≥ 1500gm < 2500 gm) neonates constituted 8 (10.4%) and 20 (26.0%) respectively. Among maternal risk factors, prolonged rupture of membrane (PROM) was most common 11 (14.3%). Among intervention, mechanical ventilation was done in 33 (42.9%) and the umbilical vein catheterisation was done in 36 (46.8%) neonates (Table 1).

**Table 1:** General characteristics of culture-positive neonates and maternal risk factors.

Variables	EONS	LONS	Frequency n (%)
**Gender**			
Male	34	20	54 (70.1)
Female	17	16	33 (29.9)
**Gestational age**			
Preterm	15	4	19 (24.7)
Term	36	22	58 (75.3)
**Birth weight (Gram)**			
< 2500	21	7	28 (36.4)
> 2500	30	19	49 (62.3)
**Mode of delivery**			
Vaginal	28	16	44 (57.1)
Caesarean section	23	10	33 (42.9)
**Place of delivery**			
Inborn	12	2	14 (18.2)
Outborn	39	24	63 (81.2)
Apgar < 6 at 5 minutes of life	11	0	11 (14.3)
**Maternal variables**			
Fever	2	0	2 (2.6)
PROM (>18 hours)	10	1	11 (14.3)
Urinary tract infection	8	1	9 (11.7)
Foul discharge	2	1	3 (3.9)
**Intervention**			
Mechanical ventilation	27	6	33 (42.9)
Inotrope support	17	4	21 (27.3)
Umbilical venous catheter	31	5	36 (46.8)
Blood transfusion	16	4	20 (26.0)
Partial exchange transfusion	1	0	1 (1.3)
Mortality	8	2	10 (13.0)

The most common clinical manifestation was tachypnea/distress in 62 (80.5%) followed by poor cry 41 (53.2%), poor suckling 28 (36.4%) and temperature instability 25 (32.5%). Total leukocyte count (<4000/ mm3), absolute neutrophil count (<1800/mm3), C-reactive protein (>6), thrombocytopenia (<150000/ mm3), and hypoglycemia (<40 mg/dl) was recorded in 3 (3.9%), 7 (9.1%), 55 (71.4%), 35 (45.5%), and 8 (10.4%) respectively. The mean duration of NICU stay was 12±5.6 days.

A total of 841 specimens (blood 687, cerebrospinal fluid 51, urine 44, tracheal aspirate 48 and pus 11) were processed for culture from which, 84 (10.0%) bacteria were isolated. Most of the bacteria were isolated from blood samples 53 (63.1%), followed by tracheal aspirates 18 (21.4%), urine 6 (7.1%), pus 5 (6.0%) and cerebrospinal fluid 2 (2.4%). Gram-negative bacilli 76 (90.5%) were the predominant isolates of which, Acinetobacter baumannii was the most common 27 (32.1%). Among Gram-positive, Staphylococcus aureus was the most common 5 (6.0%) isolate (Table 2).

**Table 2:** Bacterial isolates and its distribution.

Gram negative bacteria	n (%)
A. baumannii	27 (32.1)
K. pneumoniae	16 (19.0)
Enterobacter spp.	14 (16.7)
P. aeruginosa	12 (14.3)
E. coli	3 (3.6)
K. oxytoca	2 (2.4)
Citrobacter spp.	1 (1.2)
Proteus spp.	1 (1.2)
**Gram-positive bacteria**	
S. aureus	5 (6.0)
^*^CONS	1 (1.2)
Enterococcus spp.	1 (1.2)
Streptococcus spp.	1 (1.2)
Total	84 (100)

* CONS: Coagulase negative Staphylococcus

**Figure 1:**
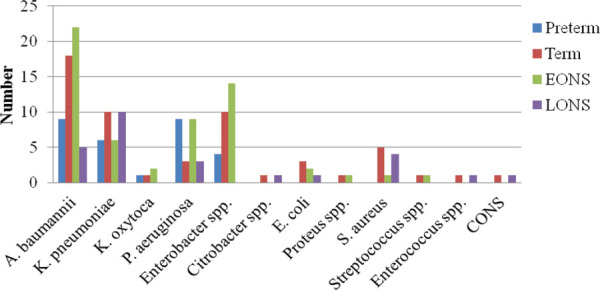
Distribution of isolates based on gestational age at birth and onset of symptoms.

Among isolates, Enterobacter spp. was common in EONS while K. pneumoniae and S. aureus were common in LONS (Figure 1).

Gram negative bacteria showed high sensitivity to Colistin 40 (100%), Meropenem 71 (94.7%), Imipinem 35 (92.1%), Polymixin B 33 (91.7%), Tigecycline 29 (87.9%), Piperacillin/Tazobactum 8 (88.9%), Fluoroquinolones: Levofloxacin 60 (83.3%), Ofloxacin 56 (77.8%) and Chloramphenicol 7 (70.0%); while high resistance to Ampicillin 74 (97.4%), Ampicillin/ Sulbactum 45 (78.9%), Azithromycin 45 (93.8%), Cephalosporins: Cefotaxime 55 (72.4%), Ceftriaxone 55 (74.3%), Cefepime 24 (66.7%), and Aminoglycosides: Gentamicin 44 (59.5%), Amikacin 41 (53.9%).

Gram positive bacteria showed high sensitivity to Vancomycin 8 (100%), Carbapenems: Meropenem 7 (100%), Imipinem 3 (75%), Levofloxacin 4 (100%), Amikacin 6 (75.0%); while high resistance to Cephalosporins: Cefotaxime 6 (75.0%), Ceftriaxone 5 (71.4%), Ampicillin 4 (57.1%), Ampicillin/Sulbactum 4 (57.1%), Azithromycin 5 (62.5%), and Colistin 2 (66.7%) (Table 3).

**Table 3:** Antibiotic sensitivity pattern of major isolates.

Antibiotics	A. baumannii S^*^/(S + R^†^) (%)	Enterobacter spp. S/(S + R) (%)	K. pneumoniae S/(S + R) (%)	P. aeruginosa S/(S + R) (%)	E. coli S/(S + R) (%)	K. oxytoca S/(S + R) (%)	S. aureus S/(S + R) (%)
Ampicillin	1/27 (3.7)	0/14 (0)	0/16 (0)	0/12 (0)	1/3 (33.3)	0/2 (0)	3/5 (60)
Ampicillin/Sulbactum	10/19 (52.6)	1/13 (7.7)	0/15 (0)	1/8 (12.5)	NT^‡^	0/2 (0)	3/5 (60)
Cefotaxime	6/27 (22.2)	2/14 (14.3)	2/16 (12.5)	7/12 (28.2)	3/3 (100)	0/2 (0)	1/5 (20)
Ceftriaxone	6/27 (22.2)	2/14 (14.3)	1/16 (6.3)	6/12 (50)	3/3 (100)	0/2 (0)	2/5 (40)
Cefoperazone/Sulbactum	14/17 (82.4)	0/8 (0)	0/11 (0)	8/8 (100)	NT	½ (50)	NT
Cefepime	7/15 (46.7)	1/7 (14.3)	2/7 (28.6)	1/5 (20)	NT	½ (50)	2/2 (100)
Cotrimoxazole	16/27 (59.3)	2/14 (14.3)	7/15 (46.7)	3/12 (25)	3/3 (100)	0/2 (0)	1/1 (100)
Gentamicin	17/27 (63)	1/14 (7.1)	2/16 (12.5)	7/12 (58.3)	3/3 (100)	0/2 (0)	3/5 (60)
Amikacin	16/27 (59.3)	1/14 (7.1)	2/16 (12.5)	11/12 (91.7)	3/3 (100)	0/2 (0)	5/5 (100)
Ofloxacin	20/27 (74.1)	12/13 (92.3)	9/15 (60)	11/11 (100)	2/2 (100)	0/2 (0)	1/3 (33.3)
Levofloxacin	22/27 (81.5)	12/13 (92.3)	10/15 (66.7)	11/11 (100)	2/2 (100)	½ (50)	3/3 (100)
Imipinem	13/14 (92.9)	8/9 (88.9)	7/8 (87.5)	5/5 (100)	NT	2/2 (100)	2/3 (66.7)
Meropenem	25/27 (92.6)	14/14 (100)	14/16 (87.5)	12/12 (100)	2/2 (100)	2/2 (100)	5/5 (100)
Colistin	15/15 (100)	NT	10/10 (100)	5/5 (100)	NT	2/2 (100)	1/2 (50)
Tigecycline	15/15 (100)	5/6 (83.3)	6/6 (100)	2/5 (40)	NT	1/1 (100)	2/2 (100)
Polymixin	15/15 (100)	5/6 (83.3)	7/8 (87.5)	4/5 (80)	NT	2/2 (100)	½ (50)
Piperacillin/Tazobactum	NT	NT	NT	8/9 (88.9)	NT	NT	NT
Norfloxacin	NT	NT	1/3 (33.3)	NT	3/3 (100)	NT	NT
Chloram phenicol	2/2 (100)	3/3 (100)	2/3 (66.7)	0/2 (0)	NT	NT	NT
Penicillin	NT	NT	NT	NT	NT	NT	0/5 (0)
Cloxacillin	NT	NT	NT	NT	NT	NT	2/5 (40)
Vancomycin	NT	NT	NT	NT	NT	NT	5/5 (100)
Azithromycin	1/12 (8.3)	2/13 (15.4)	0/14 (0)	0/7 (0)	NT	0/2 (0)	2/5 (40)

* S= Sensitive;

† R = Resistant;

‡ NT = Not Tested.

**Figure 2:**
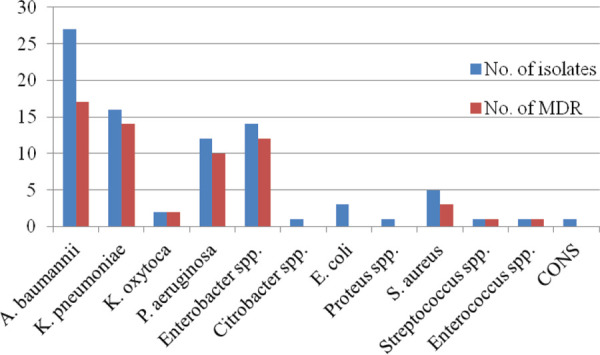
Distribution of MDR organisms.

Multidrug Resistance (MDR) is defined as acquired resistance to at least one drug from three or more antibiotic categories.^10^ Among Gram-negative bacilli, 93.0% of Enterobacter spp. demonstrated MDR while among Gram-positive bacteria, 60% of S. aureus showed MDR (Figure 2).

## DISCUSSION

Neonatal sepsis is a common cause of morbidity and mortality among neonates in NICU. The maternal risk factors, prematurity, low immunity, invasive procedures, inadequate hand hygiene may contribute to the neonatal sepsis. Our study showed the culture positivity rate of 10.0% among all clinically suspected sepsis. This finding is similar to another study conducted by Thapa et al. (10.8%).^11^ Lower positivity rate (6.2%) was elicited in a study conducted in India,^12^ whereas higher positivity rate was elicited in studies done in Kanti Children's hospital (16.9%), Patan hospital (20.7%), India (46.0%) and Egypt (42.8%).^13–16^ The variation may be due to the culture techniques, administration of antibiotics before obtaining culture specimen and study designs. In our research, the most common clinical presentation was respiratory distress which is in agreement with the different studies conducted in Nepal and Egypt.^16–18^

The present study showed EONS to be more common than LONS which accords to studies done by Thapa, et al. Pokhrel, et al. and Patel, et al.^4,11,16^ whereas contrasts to the studies done by Yadav, et al. and Shehab, et al. where LONS was more common.^15,19^ This may be because, most of the neonates with EONS in the present study were outborn (76.5%) and horizontal transmission of bacteria might have occurred from the delivery rooms, NICU rooms, during transportation or vertical transmission from mother's genital tract colonized with the pathogens.

Our study showed Gram-negative bacteria as the predominant isolates similar to recent studies done in Nepal, India, and Egypt,^11,12,14,16,20^ while Gram-positive isolates were more common in studies done in Nepal and other countries (India, Mexico, Egypt, and Norway).^13,19,21,22^ The most common isolate in our study was A. baumannii but most common organism causing LONS was K. pneumoniae. Acinetobacter baumannii was also the most common isolate in studies done by Thapa, et al. and Agarwal, et al.,^11,12^ while K. pneumoniae was isolated in studies done by Pokhrel, et al. Shrestha, et al. and Mohsen, et al.^14,16,20^ This wide variation in the occurrence of pathogens is due to the fact that they vary from place to place and also with the time of onset of illness.^23,24^

In the present work, the majority of the isolates exhibited resistance to commonly used antibiotics such as Penicillin, Cloxacillin, Ampicillin, Ampicillin/ Sulbactam, Azithromycin, Cefotaxim, Ceftriaxone, and Cotrimoxazole. The finding is in agreement with several other studies done in India, Nepal, Pakistan and Egypt.^12,16,19,25,26^

Acinetobacter baumannii being the most common isolate in our study may be the threat in the near future as the organism is highly capable of becoming multidrug-resistant and due to its property of clonal expansion.^27,28^ It has shown resistance to drugs such as Carbapenems, expanded spectrum Cephaloporins and Colistin in the studies done in India, Nepal and Egypt which is an alarming sign.^12,16,29^ However, in our study, it showed high sensitivity to the antibiotics like Colistin, Carbapenems, Levofloxacin, Tigecycline and Polymixin and moderate sensitivity to Aminoglycosides.

Klebsiella pneumoniae and Enterobacter spp. exhibited resistance to Penicillin, Cephalosporins, Aminoglycosides and Macrolides, and high sensitivity to the antibiotics such as Fluoroquinolones, Carbapenems, Colistin, Tigecycline, and Polymixin. Similar pattern of susceptibility with most of the antibiotics were also observed in other studies.^12,14,16^

Pseudomonas aeruginosa also exhibited high resistance to Penicillin, Cephalosporins, Cotrimoxazole while high sensitivity to Aminoglycosides, Fluoroquinolones, Carbapenems, Colistin and Piperacillin/Tazobactum. The pattern is different in a different study. A recent study was done in Nepal,^11^ the organism didn't show resistance to any antibiotics while another study done in Egypt,^14^ the organism demonstrated resistance pattern similar to our study.

E. coli, however, showed high sensitivity to most of the antibiotics and resistance to only Ampicillin. This finding is in contrast to the other studies where the organism showed resistance to most of antimicrobials.^14,16^

In our study, Gram-positive bacteria were isolated very less in number in comparison to other studies.^11,12,15,16^ This may be due to culture technique and administration of antibiotics to the mother before delivery. Staphylococcus aureus was most commonly isolated, of which 60.0% were methicillin-resistant S. aureus (MRSA). However, they demonstrated high sensitivity to Vancomycin, Meropenem, Levofloxacin (100%); the pattern similar to other studies.^13,16^

In the present work, MDR organisms accounted for 72.6% of all the isolates; 62.5% among Gram-positive and 73.7% among Gram-negative bacteria. This finding is comparable to studies done by Pokhrel, et al. and Agarwal, et al.^12,16^ Higher proportion of MDR was observed among EONS (77.6%) as compared to LONS (61.5%) cases. The emergence of MDR among EONS cases poses a great challenge in their treatment leading to increased morbidity and mortality.

In our study, the case fatality rate was 13.0% which is less than other studies done in Nepal, India and Egypt.^3,5,7.12,14,16^ The highest mortality was observed with K. pneumoniae (40.0%) followed by A. baumannii (30.0%) sepsis. Large proportion of mortality (80.0%) was constituted by EONS babies; may be due to prematurity and association with higher rates of MDR organisms.

The limitations of this study are that, it is single centred study with small study population, and has limited yield of some pathogens (Gram positive and anaerobes). Large scale and multi-centred study are needed to generalise the findings.

## CONCLUSIONS

The increasing trend of resistance to commonly used antimicrobials has created difficulties in the treatment of neonatal sepsis. Rational use of antibiotics is mandatory to prevent the emergence of MDR organisms. For this, updated knowledge of prevalent organisms in the local hospital setting and their antibiotic sensitivity pattern is of paramount importance. This will help clinicians to formulate local guidelines and strategies for the effective and timely treatment of neonatal sepsis and hence to prevent further morbidity and mortality.
